# Pathways for scaling up public health interventions

**DOI:** 10.1186/s12889-017-4572-5

**Published:** 2017-08-01

**Authors:** Devon Indig, Karen Lee, Anne Grunseit, Andrew Milat, Adrian Bauman

**Affiliations:** 1 The Australian Prevention Partnership Centre and School of Public Health, University of Sydney, Sydney, Australia; 2 School of Public Health, University of Sydney, Sydney, Australia; 3 Centre for Epidemiology and Evidence, New South Wales Ministry of Health, 73 Miller Street, North Sydney NSW, Sydney, 2060 Australia

**Keywords:** Scaling up, Public health interventions, Program implementation

## Abstract

**Background:**

To achieve population-wide health improvement, public health interventions found effective in selected samples need to be ‘scaled up’ and implemented more widely. The pathways through which interventions are scaled up are not well characterised. The aim of this paper is to identify examples of public health interventions which have been scaled up and to develop a conceptual framework which quantifies and describes this process.

**Methods:**

A multi-stage international literature search was undertaken to identify examples of public health interventions in high income countries that have been scaled up or implemented at scale. Initial abstract review identified articles which met all the criteria of being a: 1) public health intervention; 2) chronic disease prevention focus; 3) program delivered at a wide geographical scale (state, national or international). Interventions were reviewed and coded into a conceptual framework pathway to document their scaling up process. For each program, an in-depth review of the identified articles was undertaken along with a broad internet based search to determine the outcomes of the dissemination process. A conceptual framework of scaling up pathways was developed that involved four stages (development, efficacy testing, real world trial and dissemination) to which the 40 programs were mapped.

**Results:**

The search identified 40 public health interventions that showed evidence of being scaled up. Four pathways were identified to capture the different scaling up trajectories taken which included: ‘Type I – Comprehensive’ (55%) which passed through all four stages, ‘Type II – Efficacy omitters’ (5%) which did not conduct efficacy testing, ‘Type III – Trial omitters’ (25%) which did not conduct a real world trial, and ‘Type IV – At scale dissemination’ (15%) which skipped both efficacy testing and a real world trial.

**Conclusions:**

This is the first study to classify and quantify the potential pathways through which public health interventions in high income countries are scaled up to reach the broader population. Mapping these pathways not only demonstrates the different trajectories that occur in scaling up public health interventions, but also allows the variation across scaling up pathways to be classified. The policy and practice determinants leading to each pathway remain for future study, especially to identify the conditions under which efficacy and replication stages are missing.

## Background

In order to achieve population-wide health improvement, public health interventions found effective in a controlled research setting should be ‘scaled up’ and implemented more widely [1, 2]. Scaling up refers to *“deliberate efforts to increase the impact of successfully tested health interventions so as to benefit more people and to foster policy and program development on a lasting basis”* [2]. The scalability of an intervention is determined by its effectiveness and by the likely reach and adoption of the intervention, the costs of operating at larger scale and the acceptability and fit of the intervention within the local policy context [3]. Unfortunately, the majority of published public health research remains focused on describing patterns of risk and disease, or describing results of controlled trials in highly selected samples, rather than providing evidence of the effectiveness of interventions at the population-level [4].

There is a growing literature describing operational frameworks for scaling up health interventions [2, 5–13]. These frameworks focus on providing advice to policy-makers and funding agencies (mostly from low and middle income countries) about the steps needed to scale up an intervention. For example, in the framework developed by Milat (2014), the operational steps included: 1. Assessing whether the intervention is scalable; 2. Developing a scale up plan; 3. Securing resources and preparing for scaling up; and 4. Scaling up the intervention using the developed plan [10, 11]. Though operationally useful, these frameworks remain mostly theoretical with a limited number of case studies of best practice. There were no papers found which classified the scaling up pathways that were followed for a diverse range of real world program examples.

The aim of this paper is to review the literature to identify examples of public health interventions in high income countries that have been scaled up and to use them to develop a classification system to describe the pathways taken from program development to population-wide dissemination. We then apply this classification system to a sample of public health programs that have been scaled up to characterise the scaling up stages that take place in real world settings.

## Methods

### Literature review search strategy

We conducted a search of the peer review literature for examples of public health interventions focusing on the prevention of chronic diseases which have been scaled up. It should be noted that this literature search was not for the purpose of conducting a systematic review as such, but rather to identify indicative studies of programs which have been scaled up. The terminology used to identify potential examples of scaled up interventions included reference to ‘scale’, ‘dissemination’, ‘diffusion’, among other terms described in Table 1. Studies were included if they were published in English and conducted among humans from January 1990 to December 2014. A summary of the databases searched and search terms used are outlined in Table 1.

**Table 1:** Literature search criteria

Databases used	Medline, Embase, Informit
Search terms included	1. Area of focus: (Health promotion OR Public health OR Primary Prevention OR Secondary prevention) AND 2. Activity: (Scalability OR Scale up OR Scaling up OR Adoption OR Translational research OR Dissemination OR Interventional /Intervention research OR Diffusion) AND 3. Condition type: (Cardiovascular OR Diabetes Mellitus OR Physical Activity OR Obesity OR Nutrition OR Diet OR Smoking OR Smoker OR Smoke OR Exercise OR Physical Inactivity OR Overweight OR Chronic Disease OR Non-communicable disease)
Search key areas excluded	Cancer, HIV, Maternal and Child health, Developing countries

**Fig. 1:**
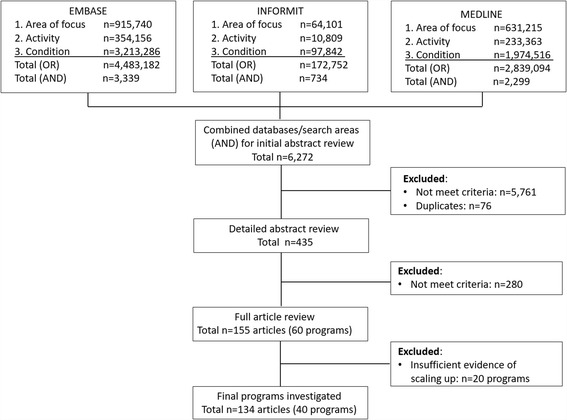
Literature search results

The literature review was conducted in June 2015. The initial search across the three databases and three search areas (area of focus, activity and condition type combined with ‘OR’) identified 7,495,029 and 6272 articles (combined with ‘AND’), respectively (see Fig. 1). As the current evidence base is largely based on programs scaled up in low and middle income countries our review focused on chronic disease prevention programs in high income countries and studies that addressed scale up of cancer, HIV, maternal and child health and studies conducted in low income countries were excluded. The second stage involved an abstract review to determine which articles met all the criteria of being a: 1) public health intervention; 2) chronic disease prevention focus; 3) program delivered at a wide geographical scale (state, national or international). This review resulted in the exclusion of 5761 articles and a further 76 duplicates, resulting in 435 articles. A more detailed abstract appraisal left 155 articles, representing 60 individual programs that were further investigated for evidence of being scaled up and full review.

### Evidence of scaling up

For each of the 60 of potentially scaled up programs identified, a detailed review of the associated papers was undertaken to determine the steps taken in program development, implementation and dissemination. This included reviewing other published articles on the same program (usually on prior pilot/efficacy studies) followed by a broad internet based search to determine the outcomes/end result of the dissemination process. The internet-based search was conducted on Google and/or Google scholar, using key words such as the: program name; chief investigator(s), program developer, author’s name; and the funding or supporting agency or institution name (if known or relevant). If no further evidence of scaling up of programs identified in the initial review was found through these means, the program was flagged as ‘outcome unknown’. Where websites were in languages other than English, the ‘Google translate’ feature was employed to determine the extent to which the program was still operational or not. This detailed review and search excluded a further 20 programs either because there was insufficient evidence of scaling up or there was evidence that scaling up had not occurred; 40 programs were therefore fully investigated for the pathways taken to scaling up.

### Documenting program pathways

The remaining 40 programs were used to characterise the stages from public health program development through to dissemination. From this, a conceptual framework comprising four stages was developed to describe the scaling up process. Each program was mapped to this framework and common pathways were summarised. The characteristics of each program (including the program focus and target population) and documented outcomes were also captured where they could be identified. The outcomes used were derived from Bauman & Nutbeam [14] and included:

- *Institutionalised*: evidence that a program had been successfully diffused into a community and integrated into the long-term functions of the host agency or organisation.
- *Commercialised*: evidence of program developers entering into partnership with private entities and/or program materials are available for purchase or can be implemented with financial reimbursement.
- *Adapted*: evidence that the intervention has been adapted or customised for a different population or sub-group.
- *Unknown*: no further information available on the outcomes of the intervention.

It should be noted that these outcomes were not mutually exclusive as some programs, particularly ones that were implemented in more than one country, fit the definition of more than one category. It should also be noted that these outcomes were mapped using publicly available information so may be incomplete or not up to date. In particular, if a program changed names, it was challenging to determine if it was the same program.

### Development of the conceptual framework

**Fig. 2:**
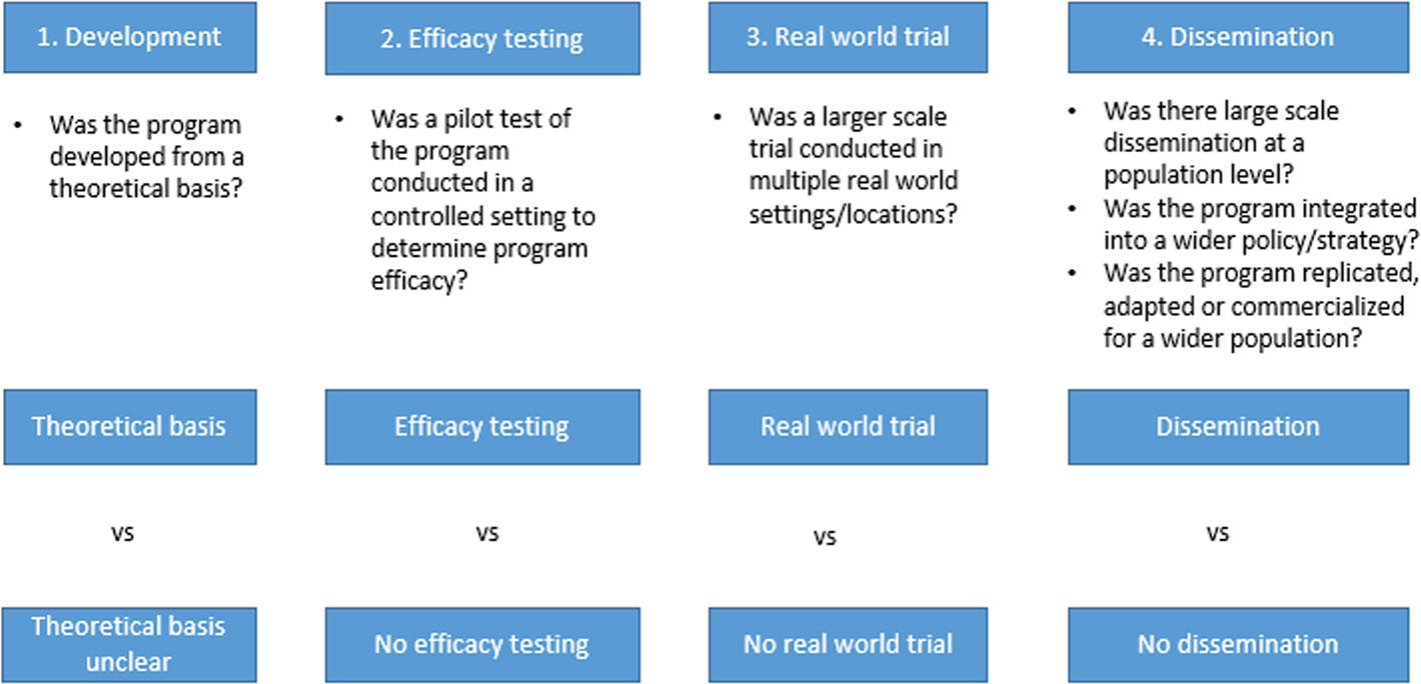
Scaling up stages

Four primary stages of scaling up were adapted from the public health research translation model for building evidence for public health programs [14] and are outlined in Fig. 2. The first stage identifies the program development process, including whether or not it had a theoretical basis. The second stage identifies whether or not the program underwent pilot testing or was tested in a controlled setting to determine program efficacy. The third stage examines whether the scaling up process included the implementation of a real world trial of the program across multiple settings and locations, which is also described as field testing or replication [14]. The final stage identified whether the program was disseminated at a population level, including whether it was adapted, institutionalised or commercialised. Note that a program may have undergone refinement to improve its effectiveness for the target population or setting at any point throughout the four-stage scaling up process. This framework is also similarly aligned to Barker’s framework for scaling up interventions in Africa which proposes four stages including 1) Set up; 2) Develop scalable unit; 3) Testing; 4) Go to full-scale [12].

## Results

### Program description

Table 2 provides an overview of the 40 programs identified in this review as being scaled up. It should be noted that these programs do not represent a comprehensive list of all public health intervention programs that have been scaled up, but a sample of chronic disease prevention studies that provide reliable evidence of being scaled up. The majority of the programs (70%) were from the United States, followed by Australia (18%). The primary target populations of the interventions were children and adolescents (43%), followed by whole population approaches (23%). Program focus was dominated by physical activity programs (38%), followed by nutrition (28%), health promotion (23%) and obesity prevention more broadly (23%).

**Table 2:** Scaled up program description

Program Name	Program focus	Target population	Country	Scaling up pathway^a^	Outcome
5 A day Power Plus [24]	Nutrition	Schools/<18 years	USA	III	Unknown
Action Schools! BC [15]	Multiple: Physical activity/nutrition	Schools/<18 years	Canada	I	Institutionalised
Active Living Every Day [25]	Physical activity	Whole population	USA	I	Commercialised
Ageing Well and Healthily [26]	Multiple: Health promotion/physical activity	Older adults	Netherlands	I	Adapted
Ask-Advise-Refer [27]	Smoking cessation	Whole population	USA	I	Institutionalised
Body & Soul [28]	Nutrition	Other: Faith-based minority groups	USA	I	Unknown
Child and Adolescent Trial for Cardiovascular Health (CATCH) [29]	Health promotion	Schools/<18 years	USA	I	Institutionalised & Commercialised
Community Health Activities Model Program for Seniors (CHAMPS) [30]	Physical activity	Older adults	USA	I	Institutionalised & Adapted
Coronary Health Improvement Project (CHIP) [31]	Health promotion	Other: People at-risk of cardiovascular disease	Multiple: USA, Canada	I	Commercialised
Choice, Control & Change [32]	Multiple: Nutrition/obesity prevention	Schools/<18 years	USA	III	Commercialised
COACH APPROACH [33]	Physical activity	Whole population	USA	I	Commercialised
Color Me Healthy [34]	Multiple: Physical activity/nutrition	Schools/<18 years	USA	IV	Commercialised
Diabetes Prevention Program (DPP) [35]	Health promotion	Other: People at-risk of diabetes	Multiple: USA, Australia, UK	I	Institutionalised & Adapted
Dutch Obesity Intervention in Teenagers (DOiT) [36]	Multiple: Health promotion/obesity prevention	Schools/<18 years	Netherlands	III	Unknown
Exercise Your Options [37]	Multiple: Physical activity/nutrition	Schools/<18 years	USA	IV	Institutionalised
Fit WIC [38]	Obesity prevention	Schools/<18 years	USA	III	Institutionalised
Fun 5 [39]	Multiple: Physical activity/nutrition	Schools/<18 years	USA	III	Institutionalised
Get Healthy Information and Coaching Service [40]	Health promotion	Whole population	Australia	I	Institutionalised
Good Ageing in Lahti Region (GOAL) Lifestyle Implementation Trial [41]	Health promotion	Other: People at-risk of diabetes	Multiple: Finland, Australia	I	Unknown
Guided Supermarket Tours [42]	Nutrition	Whole population	Netherlands	II	Unknown
Healthy Together [43]	Health promotion	Whole population	Australia	IV	Institutionalised
Hip-Hop to Health [44]	Physical activity	Other: Minority children	USA	I	Commercialised
JUMP-in [45]	Multiple: Physical activity/obesity prevention	Schools/<18 years	Netherlands	III	Unknown
Lighten Up to Healthy Lifestyle [17]	Obesity prevention	Whole population	Australia	III	Institutionalised
Melbourne Diabetes Prevention Study [46]	Health promotion	Other: People 50+ years at risk of diabetes	Australia	I	Institutionalised
Mighty Moves [47]	Multiple: Physical activity/nutrition	Schools/<18 years	USA	III	Commercialised
Mind, Exercise, Nutrition…Do it! (MEND) [48]	Health promotion	Schools/<18 years	Multiple: USA, Australia, UK	I	Institutionalised & Adapted
MOVE! Weight management program for Veterans [49]	Obesity prevention	Other: Veterans	USA	III	Institutionalised
Not on Tobacco (N-O-T) [50]	Smoking cessation	Schools/<18 years	USA	I	Institutionalised
Nutrition for Life [51]	Nutrition	Schools/<18 years	USA	IV	Unknown
Planet Health [52]	Obesity prevention	Schools/<18 years	USA	I	Institutionalised
Project Energize [16]	Multiple: Physical activity/obesity prevention	Schools/<18 years	New Zealand	II	Institutionalised & Adapted
Shaping Up My Choices [53]	Nutrition	Schools/<18 years	USA	I	Institutionalised
Sports, Play, and Active Recreation for Kids (SPARK) [54]	Physical activity	Schools Schools/<18 years	USA	I	Institutionalised
Staying Free [55]	Smoking cessation	Other: Smokers in acute care settings	Multiple: USA, Canada	I	Adapted
Strong for Life [56]	Physical activity	Older adults	USA	I	Commercialised
StrongWomen Program [18]	Physical activity	Other: Women (40+ years)	USA	IV	Institutionalised
Take 10! [57]	Physical activity	Schools/<18 years	USA	III	Institutionalised
Txt2Stop [58]	Smoking cessation	Whole population	UK	I	Unknown
Walk Kansas [59]	Physical activity	Whole population	USA	IV	Institutionalised

^a^Scaling up pathways - I: Comprehensive; II: Efficacy omitter; III: Trial omitter; IV: At scale dissemination

### Scaling up pathways

**Fig. 3:**
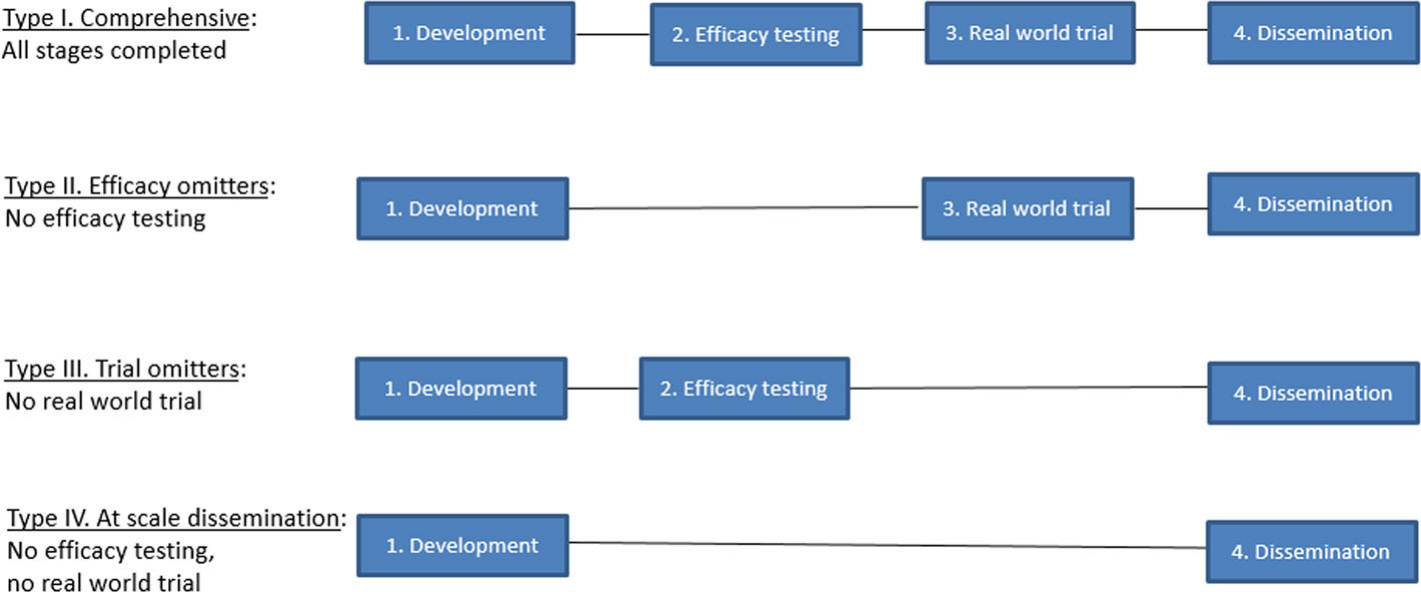
Scaling up pathways

Each of the 40 programs were mapped against the four stages described above to describe the trajectory or pathway taken towards scaling up. For inclusion, all programs had to have evidence of stage 1 (Development) and stage 4 (Dissemination). A summary of the main scaling up pathways is provided in Fig. 3. Each pathway is subsequently described further with examples.

### Type I – Comprehensive

**Fig. 4:**
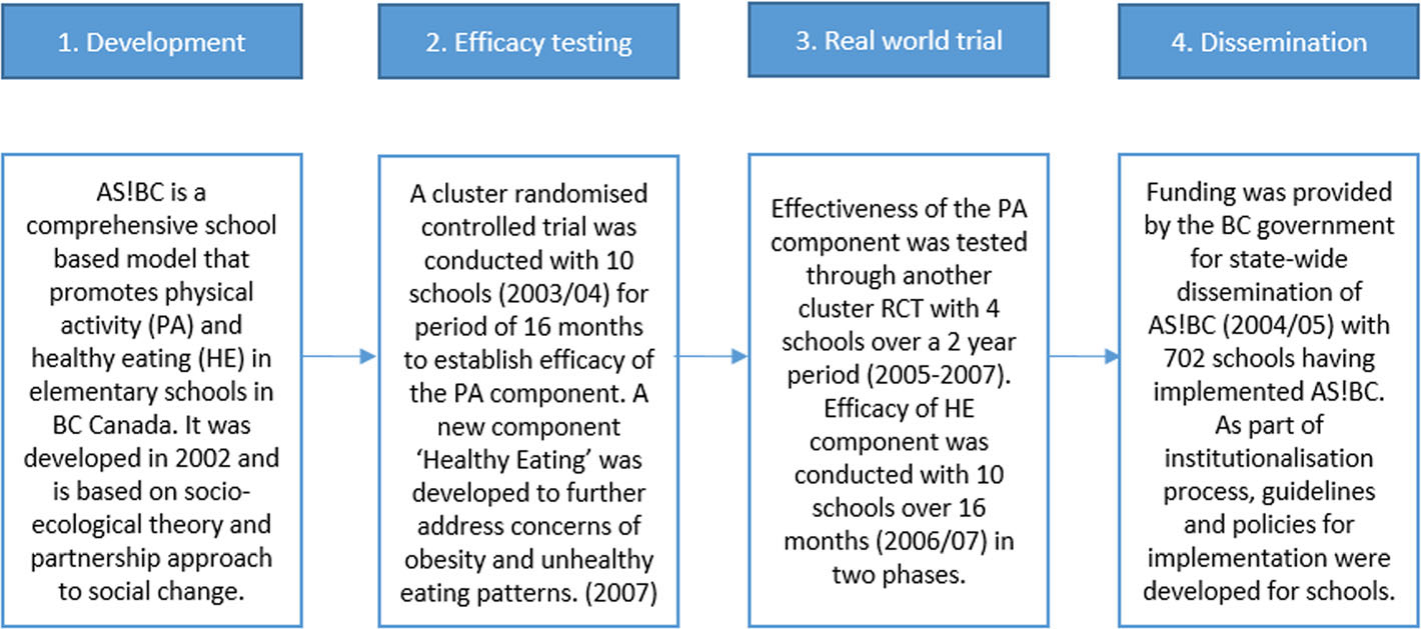
Comprehensive case study: *Action! Schools BC, Canada*

The most common pathway for the 40 programs was ‘Type I – Comprehensive’ involving adhering to all four stages of scaling up which accounted for 55% of the programs. An example of a program that followed the ‘Type I – Comprehensive’ pathway is the *Action! Schools BC* physical activity and healthy eating program from Canada [15]. As described in Fig. 4, all stages of the scaling pathway were adhered to in the development and dissemination of this program. All scaled up programs that had been implemented in more than one country (*n* = 5) followed the comprehensive pathway.

### Type II – Efficacy omitter

**Fig. 5:**
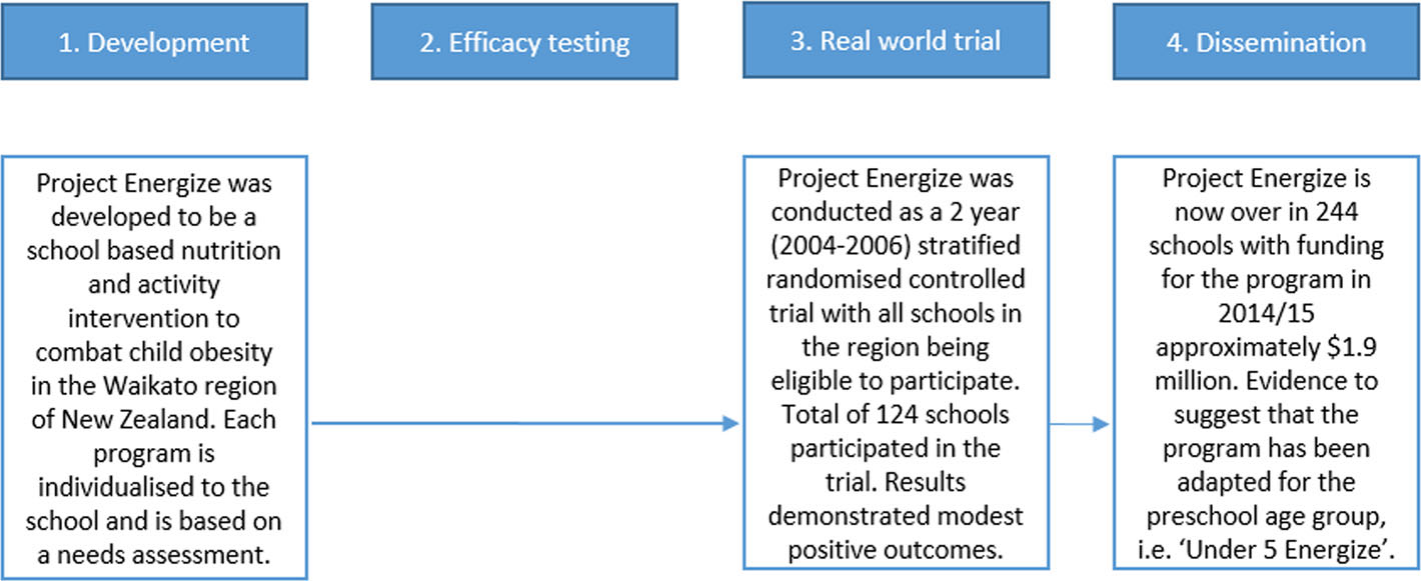
Efficacy omitters case study: *Project Energize, New Zealand*

Only two programs (5%) out of the 40 identified did not have documented evidence of any efficacy testing step, which we have referred to as ‘Type II – Efficacy omitters’. An example of this scaling up pathway was *Project Energize* from New Zealand (Fig. 5) [16]. For these two programs, there was also no evidence of program refinement.

### Type III – Trial omitter

**Fig. 6:**
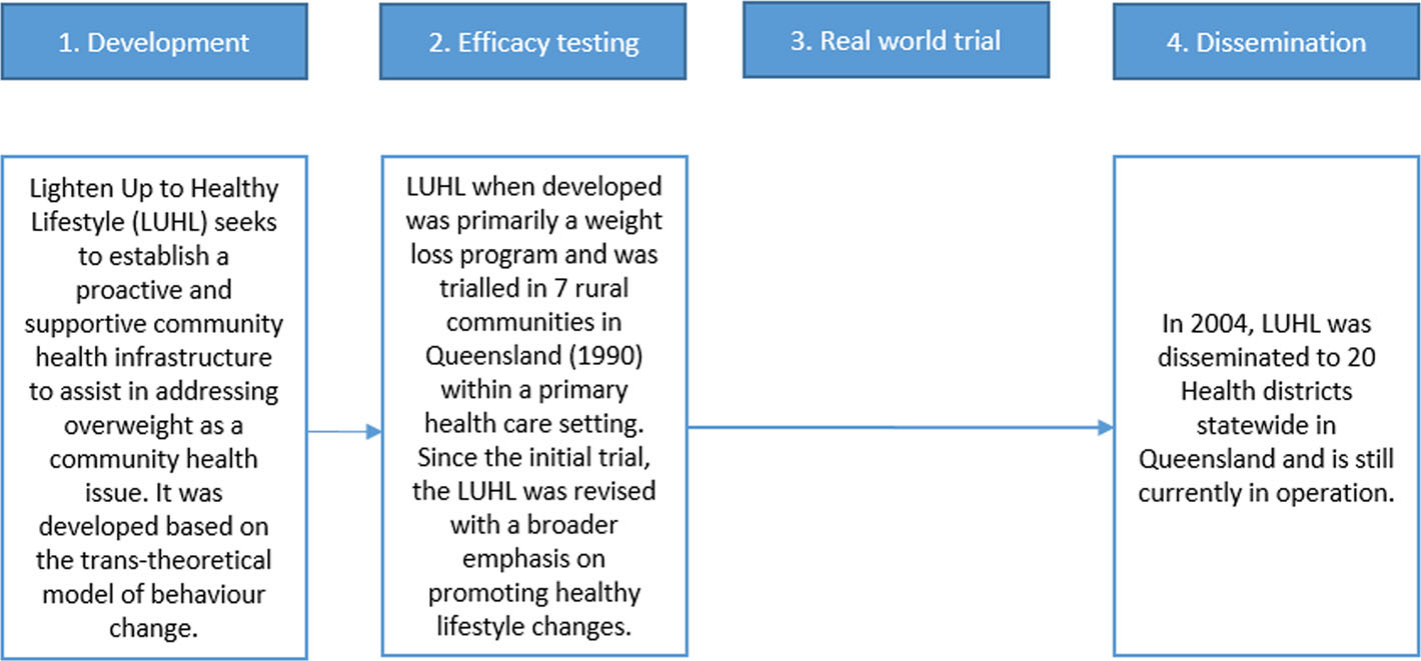
Trial omitters case study: *Lighten Up to Healthy Lifestyle, Australia*

One in four (25%) of the 40 scaled up programs were categorised as ‘Type III – Trial omitters’ where a real world trial was not conducted. *Lighten Up to Healthy Lifestyle* is an example of a program which appeared to skip the real world trial stage of scaling up (Fig. 6) [17]. Programs which fitted this description were more likely than other scaled up pathways to have multiple program focus areas (such as physical activity and nutrition) and to focus on youth populations.

### Type IV – At scale dissemination

**Fig. 7:**
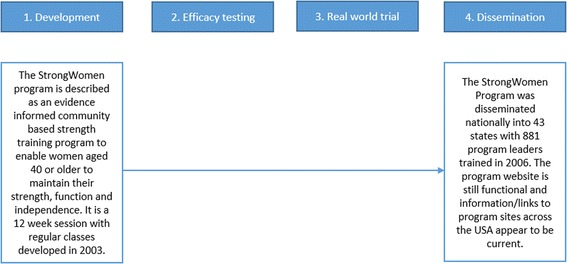
At scale dissemination case study: *StrongWomen program, USA*

‘Type IV - At scale dissemination’ involved programs that proceeded directly from development to dissemination, skipping both efficacy testing and real world trials, and typified 15% of programs. The *StrongWomen Program* is an example of a program which appeared to follow this trajectory (Fig. 7) [18].

### Outcomes

The final outcome of the dissemination process resulted in 55% (*N* = 22) of the programs being institutionalised (4 of which were also adapted for specific populations and 1 also commercialised), 20% (*N* = 8) of the programs being commercialised, a further 5% (*N* = 2) were adapted and the remaining 20% (*N* = 8) had an unknown outcome. There were no differences in the outcomes when reviewed for each scaling up pathway (see Table 3 below). Other program characteristics, such as country of implementation, were more strongly associated with outcome; for example, 100% of programs that were commercialised had been implemented in the United States.

**Table 3:** Scaled up program pathways by outcomes

Outcome	Type I Comprehensive % (N)	Type II Efficacy omitter % (N)	Type III Trial omitter % (N)	Type IV At scale dissemination % (N)	Total (all programs) % (N)
Institutionalised	36.4 (8)	0.0 (0)	50.0 (5)	66.7 (4)	42.5 (17)
Institutionalised & Adapted	13.6 (3)	50.0 (1)	0.0 (0)	0.0 (0)	10.0 (4)
Institutionalised & Commercialised	4.5 (1)	0.0 (0)	0.0 (0)	0.0 (0)	2.5 (1)
Commercialised	22.7 (5)	0.0 (0)	20.0 (2)	16.7 (1)	20.0 (8)
Adapted	9.1 (2)	0.0 (0)	0.0 (0)	0.0 (0)	5.0 (2)
Unknown	13.6 (3)	50.0 (1)	30.0 (3)	16.7 (1)	20.0 (8)
Total	100.0 (22)	100.0 (2)	100.0 (10)	100.0 (6)	100.0 (40)

Additionally, just under half (43%) of the programs had a documented refinement process such as the RE-AIM (Reach Effectiveness - Adoption Implementation Maintenance) framework [19]. RE-AIM and similar frameworks encourage program planners to examine program reach, intervention effectiveness and contextual factors that can improve the sustainable adoption and implementation of interventions. The application of the RE-AIM refinement process may have assisted these programs in achieving improved outcomes.

## Discussion

This, to the authors’ knowledge, is the first study to describe and classify the typical pathways through which a diverse range of public health interventions in real world settings are scaled up to reach the broader population. This study builds on previous research which has focused on developing guidance on best practice scale up frameworks [10–13]. The ‘Type I - Comprehensive’ four-stage evidence pathway described in our conceptual model starts with program development and moves into efficacy testing, conducting a real world trial and finally population-wide dissemination mirrors key steps widely advocated in scale up frameworks proposed in both high income [10, 11] and low-middle income contexts [12]. However, the typology developed and applied in the current study to 40 scaled up public health programs, demonstrates that the process of scaling up can be variable in its use of evidence and does not always follow a linear pathway. Many of these interventions reported using a structured refinement process, such as the RE-AIM framework [1, 12, 19] and the most common pathway, taken in over half of interventions reviewed, was described as the ‘Type I - Comprehensive’ pathway, where all the stages were adhered to. What is still unclear is whether adherence to all four stages proposed in many frameworks [10–12] results in greater success in population reach and better sustainability, with no clear association identified between scaling up pathways and program outcomes.

The scaling up pathway which occurred the least often (5% of interventions identified) was ‘Type II – Efficacy omitters’ where efficacy testing in a controlled setting did not appear to take place before a real world trial was conducted. It is possible that for these interventions, efficacy testing did take place but the findings were not published or accessible. Alternatively, it may be that funding was available to implement the program quickly in a real world trial and efficacy testing was not conducted for expediency. Whatever the underlying reason, scaling up an intervention without evidence of its effectiveness must be weighed up against the potential benefits of enabling a broader population to have access to the intervention in a real world trial.

One quarter of interventions were found to omit a real world effectiveness trial or replication studies (‘Type III – Trial omitters’), and progressed directly from efficacy testing to widespread dissemination. One explanation for this pathway is that once an intervention has been demonstrated to be effective in a controlled setting, policy-makers may wish to disseminate it widely rather than wait for the intervention to undergo another level of testing in a real world trial [3]. This can be a risky approach as intervention effects have been observed to drop from efficacy studies to effectiveness studies and drop further when implemented at scale [1]. Without testing in a real world setting, there is potential for the intervention to be less effective and decrease its potential benefits for the target population. Effectiveness studies in a real world setting enable the intervention to be assessed in a broader patient population, across a wider geographic area and in different clinical settings which can improve the external validity of the intervention [20].

Surprisingly, nearly one in seven (15%) interventions were identified as ‘Type IV – At scale dissemination’ which described where the program went directly from the development stage to populationthe-wide dissemination, omitting both evidence generating stages of efficacy testing or real world trials. Programs in this category may have been implemented rapidly due to policy pressures, causing a shortened timescale for dissemination. The pressure for policy makers to act with imperfect evidence is widely reported [3]. As a policy maker responsible for scaling public health interventions recently noted in qualitative research study that examined scale up decision making: ‘Plenty of critics were happy to say “well it’s never been done before, how do you know it will work?” That’s the reality of any large scale population based intervention. Someone has to do it first…’ [3]. This approach is not without its risks, most obvious of which is that an ineffective intervention may be scaled up. Further qualitative research would be needed to gain a better understanding of why this approach is taken by program developers and the implications for program delivery and outcomes.

Although this is presented as a four-stage model, it was clear that the final stage of “dissemination” was not static but could sometimes involve further refinement and adaptation including translation for adoption by a range of subpopulations and across different countries [21]. Just under half (45%) of programs became institutionalised [14] or embedded into routine procedures or curricula. Even the term “at scale” varied as to size; some programs were disseminated across a city or region, whereas others (such as the *MEND* school-based health promotion program) were taken up in multiple countries [22]. Hence while we believe that these characterisations form a useful framework for examining, describing and assessing the scaling up process, clearly there is much detail and variability underlying each stage and pathway through the stages not explicitly captured by the pathways.

A primary limitation of this study is that it relied upon available published information to document the trajectory a program followed for scaling up and its outcome, which may have resulted in publication bias as a result of excluding gray literature sources. Additionally, the outcome of the programs identified (institutionalised, commercialised, etc) is an indicator of the sustainability of the program and cannot be used to determine program effectiveness in achieving its public health outcomes. Articles not published in English were also excluded which may have resulted in a language bias. Efficacy testing and real world trials may have been conducted and programs widely disseminated but not published or made available externally. Further, as programs were refined, they may have changed their name or combined with other programs, making it difficult to establish program boundaries and subsequently to identify pathways of development. Further research should be conducted that includes interviews with key stakeholders in the program development and scaling up process to develop a clearer picture of the timeframes, pathways taken and impact of the scaling up pathway on the final outcome. Further, the proportions here are based on the sample of case studies extracted around chronic disease risk factor interventions, and may not apply to other scale up areas. Nonetheless, the principles and pathways are likely to be similar, and their classification provides guidance for policymakers and practitioners in characterising scaled up population-level intervention research.

This research has identified four potential pathways that a public health intervention may follow from program development to population-wide dissemination. Developing such a classification system helps researchers and policy makers alike to better understand the stages and pathways public health interventions follow in practice in scaling up. However, in order to improve the utility of this framework, the outcomes and sustainability of programs who adhere to the different pathways should be assessed to determine their relative success. This may help to inform policy-makers seeking to urgently implement programs with insufficient evidence of the risks and benefits of doing so. It would also be important to investigate whether the scaling up pathways vary by the nature of the intervention (i.e., education program, behaviour change etc.) or by the structural characteristics of the organisations funding or the methods used to implement the scaling up process. These contextual factors are an important characteristic to take into account when considering rationales and implications of scaling up pathways followed [23].

## Conclusions

The study demonstrates that the scale up of public health interventions often follows a range of pathways which are informed by differing levels of intervention evidence. Understanding these pathways contributes to a better appreciation of the role that evidence plays in the successful scale up of public health interventions. The policy and practice determinants leading to each pathway merit further study as does the relative success of these trajectories.

## Abbreviation

RE-AIM: Reach Effectiveness - Adoption Implementation Maintenance
